# The Influence of the Gut Microbiome in Paediatric Cancer Origin and Treatment

**DOI:** 10.3390/antibiotics11111521

**Published:** 2022-11-01

**Authors:** Viktória Sági, Nóra Makra, Noémi Csoszánszki, Abel Decmann, Dóra Szabó, Miklós Garami

**Affiliations:** 1 2nd Department of Pediatrics, Faculty of Medicine, Semmelweis University, 7-9 Tűzoltó Str., 1094 Budapest, Hungary; 2 Institute of Medical Microbiology, Faculty of Medicine, Semmelweis University, 4 Nagyvárad tér, 1089 Budapest, Hungary; 3 Department of Internal Medicine and Oncology, Faculty of Medicine, Semmelweis University, 2a Korányi S. Str., 1083 Budapest, Hungary

**Keywords:** paediatric, oncology, gut microbiome, chemotherapy, dysbiosis

## Abstract

Knowledge of the complexity of the gut microbiota is expanding, and its importance in physiological processes and disease development is widely studied. The aim of this review is to present the most relevant and recent research on the associations between gut microbiota and oncologic disease. Recently, a number of associations between the gut microbiome and neoplasms—regarding tumorigenesis, prognosis and therapeutic efficacy—have been reported. The effects of the gut microbiome on these processes are via the direct and indirect immunomodulating effects of bacteria. Studies have been done mainly in adult populations, where its effect on immunomodulating therapies was unambiguous. In paediatric populations, however, due to the low number of cases and the complex therapeutic approaches, there have been only a few studies. Among them, children with acute lymphoblastic leukaemia were mainly involved. Significant alterations in the abundance of certain bacteria were associated with altered therapeutic responses. Regarding solid tumours, studies with low case numbers have been reported; no significant discoveries have been described so far. In the future, studies with larger cohorts are needed in order to better understand the associations between bacteria and neoplasms and to improve prognosis in the paediatric oncologic population.

## 1. Introduction

### The Human Microbiota and Its Significance

The human microbiota can be found all over the entire human body; mucosal surfaces are the most densely populated areas. The gut microbiota consists of approximately 3 × 10^13^ bacteria; its largest portion lives symbiotically with the host [1]. This complex ecosystem develops persistently, and it begins with the vertical transmission at the time of birth, followed by the influences of environmental factors throughout one’s life [2]. Consequently, every person develops a uniquely diverse gut microbiota. This complex ecosystem could be discovered by microbial sequencing (The Human Microbiome Project) [3]. The gastrointestinal tract is the most densely colonised system in the human body. Along with bacteria, other life forms also reside in the gastrointestinal tract in the minority, such as fungi, viruses, and archaea.

A healthy interaction between the gut microbiota and the human body is necessary to maintain homeostasis. However, specific alterations in the microbiome or dysbiosis can contribute to the development of certain diseases. It takes part in numerous physiological processes, affects certain metabolic routes and immune processes, and can be found to be associated with inflammatory processes [4]. The gut microbiota interacts with the cells of the gut wall; thus, it can participate in various regulatory mechanisms [5]. There is a direct way the microbiome can function as an antigen. Indirectly, it can induce immune processes and modifications of gene expression through metabolites (e.g., short-chain fatty acids) [6].

In case this balanced system falls over, and if the composition and function of microbiota markedly change, dysbiosis develops when not only pathogenic bacteria will flourish, but metabolic processes can also be disturbed [5]. Dysbiosis can lead to numerous inflammatory processes and neoplastic or non-neoplastic diseases [6], among others; its effects in the development of endocrine or gastrointestinal diseases and neurodevelopmental disorders (Alzheimer’s disease) are widely researched [7,8,9].

**Figure 1:**
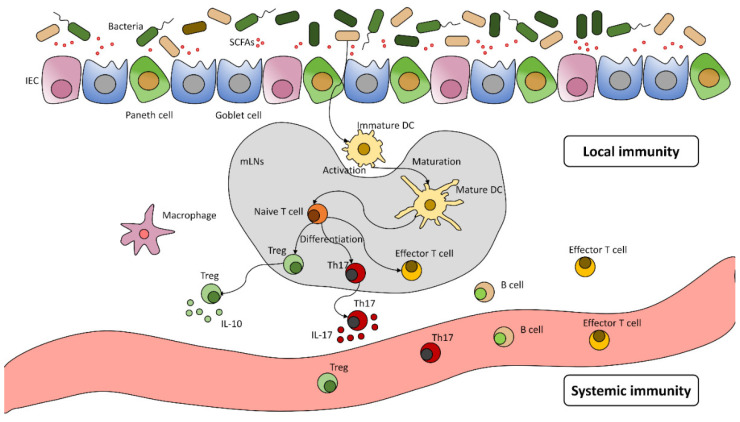
The gut microbiome and its effect on the immune system This figure is a schematic illustration of the gut microbiome affecting the immune cells. The gut microbiome could modify the immune system via direct (local lymph node reaction) and indirect ways (e.g., SCFAs). It causes a local (e.g., DC activation) and systematic response (cytokine release).

The intestinal mucosa comprises a single-layer epithelium that has intestinal epithelial cells (IECs), such as Paneth cells and goblet cells and intraepithelial lymphocytes. This unique system is in close interaction with the immune system [6]. Metabolites, such as short-chain fatty acids (SCFAs)—which can stimulate IgA secretion [10]—or directly, bacteria can activate local dendritic cells (DC), and this way, they can migrate to lymph nodes. It is the location of the T-cell activation and the induction of Treg and Th17 cells; from there, they can turn back to the gastrointestinal mucosa or the systemic circulation [11]. A local response can also develop regulatory T-cells to secrete IL-10 transforming growth factor- β (TGF β) and generate local anti-inflammatory processes [12]. Th17 cells stimulate the production of antimicrobial peptides by secreting IL-17 which results in the further release of inflammatory cytokines [11]. A systemic response also can develop: B- and T-cells enter the systemic circulation. Thus, they can react to identical antigens or cross-react to similar epitopes in different parts of the body (Figure 1) [6]. Another example of direct effect is when *Faecalibacterium prausnitzii* stimulates the differentiation of goblet cells and mucus production [13].

## 2. Microbiome and Cancer

### 2.1. Carcinogenesis

The microbiome has been suggested to contribute to carcinogenesis, with a number of possible mechanisms suggested [14]. Cell proliferation and apoptosis are strictly regulated that can be altered by the microbiome. There are microbes that can induce carcinogenesis by damaging host DNA [15,16] or by intervening in carcinogenic signalling pathways (such as Wnt/β-catenin) [17,18]. A healthy interaction between the immune system of the host and microbiota is necessary to live in symbiosis. However, when conditions change, i.e., chronic barrier breach, pathologic reactions can develop between them. Chronic inflammation that can be maintained by proinflammatory reactions induced by microbes can lead to malignancies [17,19,20]. Another way microbiome can induce carcinogenesis is by evading the immune system facilitated by *Fusobacterium nucleatum* (e.g., *F. nucleatum* protein Fap2 and human TIGIT [also called T cell immunoreceptor with Ig and ITIM domains] interaction led to the inhibition of NK cell cytotoxicity and lymphocyte cell activity in *Fusobacterium* containing tumours) [21]. Microbes play an important role in human metabolism. Toxic metabolites produced by the microbiota can also affect the immune system [14]. The role of dysbiosis in tumorigenesis is important but not yet fully discovered [22]. Dysbiosis can induce carcinogenesis and can affect the metabolism of chemotherapeutic agents [10]. Thus, learning about the gut microbiota can help us better know the diagnosis, therapy, and prognosis of neoplasms.

The relationship between the gut microbiome and tumours has been better understood in recent years, and more attention has been given to it. However, the tumour-microenvironment is still largely unknown. In particular, tumours that develop from tissues that have connections to surfaces harbouring microbiota (e.g., skin, mucosa, lung) may be affected by the local microbiome [23]. The bacteria could boost inflammation or reduce the anti-tumour response, causing the tumour to grow and spread [14,24,25]. There are still many unanswered questions about the microenvironment of tumours, such as where and how bacteria come from and get there and whether they migrate from the surrounding flora or translocate from elsewhere [23].

### 2.2. The Interaction of Gut Microbiome and Systemic Treatments

A number of drugs used to treat cancer, e.g., chemotherapy, radiotherapy and immunotherapy, have been reported to interact with the gut microbiome and, in some cases, be dependent on its composition. This has been most elegantly described in the case of irinotecan, with its second pass metabolism resulting in high luminal concentrations of its inactive metabolite, SN38G. Exposure to beta glucuronidating bacteria then re-activates SN38, resulting in high luminal concentrations. While this mechanism is unique to irinotecan, increasing evidence suggests that the gut microbiome is important in the potency and efficacy of other cancer drugs [26].

The gut microbiome has significant importance in terms of anti-tumour therapy. Certain members of the gut microbiota can affect the response given to immune checkpoint inhibitors, anti-PD-1/PD-L1, or anti-CTLA-4 therapy. Mice that have microbiomes with different compositions reacted differently to PD-1/PD-L1 blockade therapy. The assessment of gut microbiomes showed that slow tumour progression and better response to anti-PD-1 therapy could be observed in mice with a significantly higher relative abundance of *Bifidobacterium* species [27]. It could be hypothesised that a change in the gut microbiome could improve the efficacy and reduce the toxicity of certain chemotherapeutic agents. It is known that oncological therapies have effects not only on the host body but on bacteria as well; their relevance is under debate.

**Figure 2:**
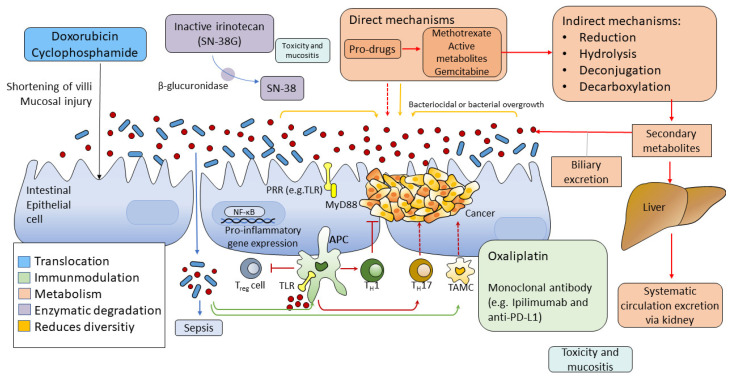
The microbiome can change the efficacy and toxicity of chemotherapeutic agents. TIMER is an acronym for the effects of chemotherapy on the gut microbiome. ‘T’ stands for translocation: bacteria migrate through the gut wall into the lymphatic system or the systemic circulation. ‘I’ stands for immunomodulation: gut microbiome might enhance chemotherapy-induced immune responses. ‘M’ stands for metabolism: the microbiome has direct and indirect effects on the effects of chemotherapy; it might strengthen or reduce the desired effects or promotes the release of toxic compounds. ‘E’ stands for: enzymatic degradation: gut microbiome has a wide range of enzymes capable of creating metabolites that can cause undesired side effects. ‘R’ stands for reduced diversity: chemotherapy can be a cause of dysbiosis. (Figure was based on the work of Alexander, J. L. et al. [28].

Although there is a rising number of studies and articles concerning the association between microbiome and neoplasms, at the moment, we know little about the mutual effects of the microbiome and pharmacological interactions in the human body [28]. Chemotherapy and radiotherapy can both cause dysbiosis. This disturbance in the composition of the microbiome might attenuate the response given to therapy and augment the chemotherapeutic toxicity (Figure 2) [28].

The other way is the translocation of bacteria, e.g., cyclophosphamide therapy can generate shortening of the intestinal villi, destroying the mucosal barrier, making way for bacteria to get through reaching secondary lymphoid organs, thus affecting the efficacy of therapy: by immunomodulation and raising the probability of developing sepsis [29]. Through the immunomodulation triggered by this method, the gut microbiota induces immune and inflammatory responses. An example of that is *Bifidobacteria,* which can modify tumour-specific T-cell induction and increase the T-cell number, thus increasing therapeutic efficacy in the tumour microenvironment in patients treated with anti-PD-L1 agents [30]. Another example is *Lactobacillus,* which can stimulate Th17 and Th1 response in patients treated with cyclophosphamide [29]. 

Articles have been published discussing how bacteria can affect therapy by altering pharmacologic agents and drug metabolism. Bacteria can cause augmentation or abolishment of desired effects or release toxic metabolites. Microbiota can perform numerous direct enzymatic processes (e.g., reduction, hydrolysis, dealkylation, dehydroxylation) that can be used in drug metabolism [26,31]. Additionally, chemotherapy can change the diversity of the microbiome by altering bile release and secondary metabolic processes [32].

The clinical studies of adults confirmed the association between gut bacteria and therapeutic efficacy. Immune checkpoint inhibitors (ICIs) are applied in patients with malignant melanoma, non-small cell lung cancer, and renal cell cancer [33,34,35]. 70–80% of cases are resistant to therapy [36,37]. Routy et al. described that overall survival and progression-free survival were better in patients that did not get prior antibiotic treatment for various reasons compared to those who have. This proves that antibiotics affect (might have a negative effect) tumour immunity and reduce immune checkpoint inhibitor therapy response. When comparing the two groups, they found that *Akkermansia muciniphila* was overrepresented at diagnosis in those who later responded better to PD-1 inhibitor treatments [27].

More studies have proved that there is an association between therapeutic efficacy and gut microbiome, although the precise mechanisms are still unknown. It is certain though that this diverse ecosystem has a positive effect on homeostasis and tumour-immune relations. Microbiome could also be used as a biomarker (prognostic factor). A detailed analysis of the gut microbiome could serve as a personalised biomarker to identify a dysbiosis-associated failure of therapy and to help find the required microbial targets that need an alteration in order to restore balance [28]. Moreover, it can help with tumour staging and determining phenotype, as we can see in the adult population in the case of *Fusobacterium,* which can be associated with a worse prognosis and stage [38].

## 3. Paediatric Specificities

The role of the gut microbiota is considered to be essential in the physiological development of the human body and the preservation of health [39]. There is a limited number of studies regarding microbiomes in the paediatric population. Numerous factors affect the development of microbiome diversity from birth. We have searched the literature (EMBASE, Web of Science Core Collection, MEDLINE, and Google Scholar; ClinicalTrials.gov) using the keywords: (gut microbiota OR gut microbiome) AND (cancer OR oncology) AND (paediatric OR children) AND (treatment OR chemotherapy). However, almost all of the studies found concern hematologic malignancies. Therefore, we aimed to review comprehensively the connection between the microbiome and oncologic treatment, mainly in this population.

**Figure 3:**
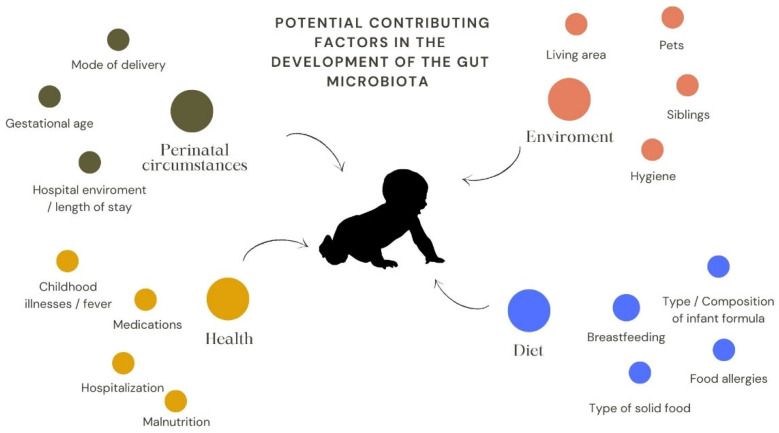
Potential contributing factors in the development of the gut microbiota. The most important factors can be grouped as perinatal circumstances, health-related, environmental and dietary factors.

It has long been thought that the intestine of neonates is sterile, and the development of the microbiota in the neonate begins at birth. However, the presence of bacteria in the meconium has altered this view [40]. The origin of the human microbiota, especially the gut microbiota, is still to be discovered, but it is suggested that the colonisation of the placenta, the amniotic fluid, and the umbilical cord might play a role [40,41]. Influencing factors are thought to be the way of delivery [42], breastfeeding and its duration [43], acquired infections and subsequent antibiotic use [44], ingested foods [45], other external and internal factors such as age, ethnicity, living area (Figure 3) [46].

Several studies have demonstrated that breastfeeding plays a crucial role in the development of gut microbiota [47]. For example, a meta-analysis has demonstrated that there is an increased risk of developing a hematologic disease in children who were not breastfed [48]. Breastfed infants have a gut microbiome rich in *Lactobacillus, Staphylococcus*, and *Bifidobacterium*, in contrast to formula-fed children. In formula-fed children, *Roseburia*, *Clostridium*, and *Anaerostipes* predominated [49]. During the introduction of solid foods to the children, the relative abundance of Bacteroidetes increases [50].

Similar to research in adults, the imbalance of the gut microbiome in late childhood could lead to immunological consequences and the development of diseases. Research has shown an association between dysbiosis and recurrent *C. difficile* infection [51], asthma [52], inflammatory bowel disease (IBD) [53], irritable bowel syndrome (IBS) [54], and metabolic syndrome [55]. Although we know more about the structure of the gut microbiome of healthy individuals, the effects of oncologic therapies such as chemotherapy and radiotherapy causing dysbiosis are still to be evaluated and described with sufficient detail, particularly in paediatric populations [56]. In the remaining part of the review, we aim to summarise available results and findings regarding the gut microbiome and its efficacy and toxicity in paediatric oncologic treatments. 

### 3.1. Microbiome and Acute Lymphocytic Leukaemia Incidence

The role of the gut microbiota is evident from as early as birth. The type of delivery is associated with different risks of malignancy. Children born via caesarean section have a higher risk of developing acute lymphocytic leukaemia (ALL). The reason is suggested to be that children born this way are not affected by the maternal vaginal microbiome, which helps them form the later gut microbiome [57]. There is, however, no association between the type of delivery and the incidence of brain cancer or lymphomas [58,59]. A meta-analysis studied the association between breastfeeding and the incidence of ALL (the most common malignancy in children). They have found that the incidence of ALL can be reduced by 14–19% if children are breastfed six or more months from birth. It is supposed that this change is due to the immunomodulating effect of the mother’s milk, by which specific nutrients, antibodies, or anti-inflammatory factors are delivered to the baby [48]. According to these and other results, the early gut microbiota is dependent on and influenced by the type of delivery, the form of feeding, hospitalisation of the newborn and the use of antibiotics. These factors might enhance or disadvantage the development of a favourable or beneficial microbiome that has a relatively higher relative abundance of *Bacteroides* and *Bifidobacterium* and lower numbers of *Clostrioides* difficile or *Escherichia coli* [60]. 

Gut microbiomes of paediatric patients suffering from ALL were studied, and it was found that at the time of diagnosis, the DNA content of the stool of patients was 29.6% lower compared to healthy individuals. The relative abundance of *Bifidobacterium*, *Lactobacillus*, and *E. coli* were significantly lower in patients with ALL [61]. It is important to note that the changes in the gut microbiome are not only directly affected by chemotherapy but also dietary modifications, antibiotic use and indirect chemotherapy effects such as immunomodulation. ALL is a severe hematologic malignancy requiring chemotherapeutic management, the minimisation of toxicity is desired, and in spite of that, high-risk patients receive aggressive therapy [62]. 

### 3.2. Treatment Induced Changes in the Microbiome

Chemotherapeutic treatments can alter the microbiome of children as it is described in a few studies mainly involving ALL patients. In the most recent literature, an association can be found regarding the paediatric population between microbiome profile and ALL and its therapy. Relative abundance of certain taxa of bacteria in the ALL population before, during and after treatment. 

A pilot study with a small cohort examined the alteration of gut microbiome before, during and after chemotherapy in children with acute lymphoblastic leukaemia (ALL). Before the initiation of chemotherapy, the gut microbiome in paediatric patients with ALL showed a larger variation between patients in comparison to healthy controls. The relative abundance of bacteria from *Bacteroidetes* phylum (esp. *Bacteroides* genu) was high until the chemotherapy was initiated, and then the relative abundance of bacteria belonging to *Bacteroides* genu decreased. After the termination of chemotherapy, recovery of the gut microbiome started; however, pre- and post-chemotherapy microbiome composition still differed after nine months in ALL patients compared to healthy controls [63].

Another study has found that alpha and beta diversity were significantly different between children with ALL and their siblings. Beta diversity alterations during chemotherapy were significantly associated with antibiotic treatment and leukaemia risk group (i.e., low or high risk). During the treatment, beneficial bacteria for health, such as *Verrucomicrobiota* phylum (especially *Akkermansia* genus), were significantly decreased [64]. A total of 199 children with ALL have been observed to have reduced microbial diversity after chemotherapy. The reduction of *Bacterioidetes*, *Faecalibacterium*, *Ruminococcaceae* and *Verrucomicrobiota* was observed, while the relative abundance of *Clostridiaceae*, *Streptococcaceae*, *Lactobacillaceae* and *Enterococcaceae* has grown [65]. These studies, however, do not provide an answer to the question of whether dysbiosis is a causative factor in tumorigenesis (or leukemogenesis) and has a modifying effect on chemotherapy or whether it is a complication of the tumorous disease and/or chemotherapy [66].

One of the results of importance in recent years was that a connection could be found between the response to oncologic therapy of paediatric solid tumours (rhabdomyosarcoma) and the patient’s microbiome diversity [67]. A pilot study reported three children with pelvic region rhabdomyosarcoma who underwent chemotherapy and radiotherapy. Despite the low case number, an association has been found between the efficacy of therapy and the microbiome composition. Alpha diversity before radiotherapy was higher in healthy controls because of the prior chemotherapy and antibiotic treatments in the disease group. Alpha diversity of patients receiving radiotherapy increased in two patients and decreased in one patient, showing no obvious effect of radiotherapy on the gut microbiome. Patients who responded better had lower microbial diversity. This study had its many limitations; however, it might lead us to a better understanding of microbiome alterations in patients receiving antitumour therapy [67]. 

### 3.3. Microbiome and Acute Complications of Chemotherapy

In the recent 50 years, the survival of paediatric haematology and oncology patients has been improved notably. One of every 800 adults is a paediatric oncological survivor [68]. These results are the effects of the intensification of the broad spectrum, systemic and combined chemotherapies. These therapies go together with various acute and chronic side effects and complications. Thus, it is of high importance to have results regarding gut microbiome and side effects of chemotherapy. 

It is reported that the gut microbiome is significantly reduced after chemotherapy compared to the pre-chemotherapy measurements. The number of some bacteria, e.g., *Bacteroides*, were significantly reduced, while others were markedly elevated, e.g., *Clostridicae* and *Streptococceae*. There was an association between the higher relative abundance of *Proteobacterium* in baseline flora at the time of diagnosis of ALL and the incidence of neutropenic fever. Also, following neutropenic fever, episodes occurred more often in patients with a higher relative abundance of *Enterococcacaea,* and diarrhoea was more frequent with higher numbers of *Streptococcaceae* [65].

Mucosal barrier injury or mucositis is a serious complication of oncologic therapies, including radio- and chemotherapy. Models that attempt to describe the pathogenesis of mucositis involve reactive oxygen species induced activation of nuclear factor kappa B (NFκB) signalling and consequential discontinuity of epithelial barrier caused by tumour necrosis factor alpha (TNFα) induced apoptosis. In this model, the gut microbiome is not a factor in the development of mucositis [69]. However, according to recent results and publications, it can be hypothesised that the gut microbiome might play a significant role in chemotherapy-induced mucositis [70,71].

### 3.4. Microbiome and Chronic Toxicity

Chemotherapies are usually toxic agents reaching the entire body, including tumourous cells and are not specific to certain tumorous cells. Most patients receiving chemotherapy live with chronic side effects and complications of therapies, both physically and psychosocially. 

The survivors of ALL, despite the success of its therapy, have medical problems [72]. They are prone to obesity and metabolic syndrome that can lead to severe complications, such as cardiovascular diseases [73,74,75]. There is a study evaluating the association between gut microbiome and obesity. It showed that dysbiosis caused by chemotherapy predisposes to complications and necessitates the prevention of obesity and metabolic syndrome in ALL survivors [76]. It seems that the number of *Faecalibacterium* that are thought to be protective was reduced [77].

Allogeneic haematopoietic stem cell transplantation (allo-HSCT) is a crucial therapeutic strategy in various malignant diseases. One of its most severe complications is graft-versus-host disease (GVHD). During GVHD, multiple organs can be affected, including the gastrointestinal tract [78]. The gut microbiota might play a critical role in this complex process. Irradiation and chemotherapy before allo-HSCT can damage the barrier integrity of the gut epithelium. Through this disrupted intestinal barrier, bacteria and their metabolites can translocate and can activate the innate immune system that can activate alloreactive donor T-cells leading to the development of GVHD [79]. Moreover, reduced bacterial diversity might lead to increased mortality in GVHD [80]. 

Patients who received chemotherapy performed inferiorly regarding executive functions, attention, concentration, processing time, and reaction time [81]. Another study claims that paediatric oncological patients who survived had 90.5% chemotherapy, from which 22.4% had post-traumatic stress disorder (PTSD), anxiety, or depression [82]. How is this associated with the gut microbiome? Regarding the gut-brain axis, in communication, endocrinological, immunological, and metabolic ways are also used [83]. There is an ongoing study that seeks answers in that context: what kind of long-term side effects have the chemotherapies regarding gut microbiome diversity in association with chronic psychologic, cognitive (anxiety, depression, PTSD) and social changes or pain? They hypothesise that if there is a reduction in the relative abundance of a bacteria—causing long-term unwanted effects—and if it was replaced, long-term complications might be avoided [84]. 

### 3.5. Interventions Targeting the Microbiome in Children

In the former sections, we have shown that the changes in the gut microbiome profile might have the predictive potential of oncologic therapy and might signal toxicity. 

In our work, we have demonstrated the significance of the change in the gut microbiome in the paediatric population regarding tumour incidence, side effects profile and the outcome of oncologic therapies. The most innovative therapeutic option is to deliberately alter the microbiome profile of paediatric oncology patients. The alteration of the gut microbiome profile of these children might prevent various acute or chronic side effects in the future. 

Prebiotics can be used to restore the ‘healthy’ gut microbiota, which are nutrients helping ‘good’ bacteria to proliferate. To enrich flora, one might give probiotics. A study of 60 children with acute leukaemia showed that the incidence of gastrointestinal side effects, such as nausea, vomiting, or meteorism, significantly decreased in patients receiving probiotics containing *Lactobacillus rhamnosus* compared to patients who have not received them [85]. Although, there are some hesitations regarding the use of probiotics. There has been a clinical study on a rather small cohort where the authors have found cases of *Lactobacillus* bacteraemia connected with probiotic intake in paediatric haemopoietic cell transplant (HCT) recipients [86]. As a way to restore a normal microbiome, faecal microbiome transplant (FMT) can also be used. It is used in *Clostridioides difficile* infections, not responding to antibiotic treatment in children with success [87]. 

## 4. Conclusions

Although studies regarding the gut microbiome are growing in number, the association between paediatric tumours, particularly paediatric solid tumours, and the gut microbiome is not well understood yet. It is difficult to study because of the relatively low number of patients (cohorts) and the difficulties in collecting samples. Also, children with solid tumours often get chemotherapy and radiotherapy, as well as intensive antibiotic prophylaxis, to prevent possible infections [88]. Of all paediatric tumours, 60% are paediatric solid tumours [89]. The most common are tumours arising from the central nervous system, neuroblastoma, soft tissue sarcoma, rhabdomyosarcoma, Wilm’s tumour, osteogenic tumours (osteosarcoma and Ewing sarcoma), and retinoblastoma. Although there are fewer histological entities in the paediatric population compared to adults, no prospective study has been designed for these neoplasms. 

Paediatric oncology patients usually receive complex and aggressive antitumor therapy, which involves surgery or chemotherapy and radiotherapy, often leading to myelosuppression. These treatments have a large effect on the whole body, including the bone marrow, liver, and gastrointestinal tract, and directly and indirectly affect the gut microbiome. The importance of the gut microbiome is confirmed by several studies regarding disease development, the efficacy of therapy, staging, or side effect manifestations. It requires more investigation, but in the future, it might be feasible to identify the microbiome profile of an individual before starting antitumor therapy in order to predict efficacy or to choose appropriate and personalised therapy. Microbiomes could also be used as a biomarker. 

It remains unanswered whether dysbiosis is a consequence or cause of neoplasms. Microbiome studies in the paediatric oncology population are limited, and associations are still unclear. More studies with larger cohorts are needed to be done in order to help develop more personalised and successful therapy in paediatric oncology.

## Data Availability

Not applicable.
